# Comparison of self-reported pain intensity, sleeping difficulty, and treatment outcomes of patients with myofascial temporomandibular disorders by age group: a prospective outcome study

**DOI:** 10.1186/1471-2474-15-423

**Published:** 2014-12-11

**Authors:** Hiroyuki Karibe, Greg Goddard, Kisaki Shimazu, Yuichi Kato, Sachie Warita-Naoi, Tomomi Kawakami

**Affiliations:** Department of Pediatric Dentistry, School of Life Dentistry, Nippon Dental University, 1-9-20 Fujimi, Chiyoda-ku, Tokyo 102-8159 Japan; Center for Orofacial Pain, University of California San Francisco, 513 Parnassus Avenue, Room S738, San Francisco, CA 94143 USA

**Keywords:** Age, Headache, Myofascial pain, Outcomes, Temporomandibular disorders

## Abstract

**Background:**

Subjective symptoms of temporomandibular disorders (TMDs) have rarely been studied by age group. We aimed to compare self-reported pain intensity, sleeping difficulty, and treatment outcomes of patients with myofascial TMDs among three age groups.

**Methods:**

The study population included 179 consecutive patients (151 women and 28 men) who underwent comprehensive clinical examinations at a university-based orofacial pain center. They were classified into myofascial pain subgroups based on the Research Diagnostic Criteria for Temporomandibular Disorders. They were stratified by age group: M1, under 20 years; M2, 20–39 years; and M3, 40 years and older. The patients scored their pretreatment symptoms (first visit) and post-treatment symptoms (last visit) on a form composed of three items that assessed pain intensity and one item that assessed sleeping difficulty. Their treatment options (i.e., pharmacotherapy, physical therapy, and orthopedic appliances) and duration were recorded. All variables were compared between sexes in each group and between the age groups by using the Kruskal–Wallis test, the Mann–Whitney *U* test, the chi-square test, and analysis of variance (p < 0.05).

**Results:**

No significant sex differences were found in any age group. Only sleeping difficulty was significantly different before treatment (p = 0.009). No significant differences were observed in the treatment options or treatment duration. After treatment, the intensity of jaw/face pain and headache and sleeping difficulty was significantly reduced in groups M2 and M3, but only the intensity of jaw/face pain was significantly decreased in group M1. The changes in the scores of pain intensity and sleeping difficulty were not different between the groups.

**Conclusions:**

Pain intensity does not differ by age group, but older patients with myofascial TMDs had greater sleeping difficulties. However, there were no differences between the age groups in the treatment outcomes. Clinicians should carefully consider the age-related characteristics of patients with myofascial TMDs when developing appropriate management strategies.

## Background

Temporomandibular disorders (TMDs) include musculoskeletal and neuromuscular conditions that involve the masticatory muscles, temporomandibular joint (TMJ), and associated structures [1]. They are a subclass of musculoskeletal disorders and cause nondental pain in the orofacial region [1]. TMDs primarily affect young and middle-aged adults rather than children or the elderly [2], although symptoms are frequently observable in the latter populations [3–6]. Most TMDs occur between 20 years and 40 years of age, show a female preponderance [7, 8], and are self-limiting or fluctuate over time [9].

TMDs are associated with many diagnostic features such as internal derangements and myogenous disorders [10]. They can be classified according to the extent of TMJ and muscular involvement. The Research Diagnostic Criteria for Temporomandibular Disorders (RDC/TMD) allow standardization and replication of research on the most common forms of TMDs [11]. Symptoms of TMDs can accordingly be investigated by diagnostic subgroups. Patients with chronic TMDs in the myofascial pain subgroup (i.e., RDC/TMD, Axis I, Group I) reportedly have greater dysfunctionality, higher pain intensity, and greater psychological difficulties, compared to patients in the intracapsular pain subgroup (i.e., RDC/TMD, Axis I, Group II or Group III) [12, 13]. In addition, young patients with myofascial pain have significantly greater difficulty in sleeping, compared to patients with TMJ-related problems [14].

Symptoms of joint-related TMDs resolve with minimal care [15]. However, symptoms of myogenous TMDs can become chronic because many patients focus less on tightness in the masticatory muscles or on the presence of trigger points, which may also be responsible for their myofascial pain. Patients with chronic myogenous TMDs may have persistent central sensitization and psychological comorbidity that is similar to patients with chronic pain [16]. Therefore, early intervention is needed to reduce muscle tenderness and associated disability. However, few studies have compared the subjective symptoms of TMDs by age group [17–19].

In this study, we aimed to compare self-reported pain intensity, sleeping difficulty, and treatment outcomes of patients with myofascial TMDs among three age groups. We hypothesized that young patients are more likely to have mild symptoms and their symptoms are easy to treat, whereas older patients are more likely to show severe symptoms, which are difficult to treat.

## Methods

### Study population

We screened 862 consecutive patients who attended the University of California–San Francisco (UCSF) Center for Orofacial Pain (San Francisco, CA), a public university-based specialty clinic that treats TMD and orofacial pain problems. We used a prospective study approach to examine pretreatment and post-treatment differences in pain intensity and sleeping difficulty in the three age groups. We obtained the necessary data from the patients’ medical records. Ethical considerations were anonymity, privacy, and obtaining informed consent from all patients. The UCSF Committee on Human Research approved this prospective outcome study.

### Diagnostic procedures

The patients underwent standardized comprehensive clinical examinations by two examiners who were diplomates of the American Board of Orofacial Pain. The examinations included provocation testing of the TMJs, measurements of the mandibular and cervical ranges of motion, determination of TMJ noise, masticatory and cervical muscle examination, cranial nerve assessment, and intraoral examination. Additional diagnostic tests (i.e., imaging, physical therapy evaluation, and other medical consultation) were performed, if needed.

The RDC/TMD guidelines were followed for classification, based on the primary diagnosis. Patients were excluded if they had neuropathic pain, generalized pain (e.g., fibromyalgia), neurovascular headache (e.g., cluster headache or migraine), or any psychiatric disorder. The RDC/TMD Axis I, Group I.a (i.e., myofascial pain) or Group I.b (i.e., myofascial pain with limited opening) included a painful response to palpation of the following muscle sites: posterior temporalis, middle temporalis, anterior temporalis, origin of the masseter, body of the masseter, insertion of the masseter, posterior mandibular region, submandibular region, lateral pterygoid area, and tendon of the temporalis [11]. Patients with clinical features fulfilling the RDC/TMD Axis I Group I.a or Group I.b criteria were classified as the myofascial pain subgroup and were the focus of this study. This myofascial pain subgroup was stratified according to age: under 20 years (group M1; n = 41); 20–39 years (group M2; n = 62); and 40 years and older (group M3; n = 76).

### Symptom measurement

The patients completed a form that assessed their symptoms at the first visit and at subsequent visits during the treatment period. In several studies, self-reported questionnaires focused on the intensity of TMD symptoms, headaches, and neck pain and related impact on activities of daily living [20–22]. Self-reported measures provide the ‘gold standard’ in assessing pain outcomes, and commonly used methods of rating pain intensity are reliable and valid [23]. Thus, the form in our study included three items related to pain intensity (jaw/face pain, headache, and neck pain) and one item related to difficulty in sleeping. To measure pain intensity and sleeping difficulty, an 11-point numeric rating scale (NRS), which ranged from 0 to 10, was used in which 0 indicated “no pain/difficulty” and 10 indicated “the worst pain/difficulty imaginable” [24]. We used the NRS because it is a well-understood measure for pain evaluation and it has an acceptable reliability [25]. The patients scored the items on the 11-point NRS by circling the number that best represented their pain intensity and sleeping difficulty. For each age group, the treatment outcomes were analyzed by comparing the scores of the first visit (i.e., pretreatment) and last visit (i.e., post-treatment). To compare the treatment outcomes between the age groups, the changes in the scores were calculated by the difference between the post-treatment and pretreatment scores (i.e., post-treatment score - pretreatment score).

### Statistical analysis

For each age group, sex differences were assessed with the Student *t* test for age and treatment duration, and by the Mann–Whitney *U* test for pain intensity and sleeping difficulty. Analysis of variance (ANOVA) and the chi-square test were used for analyzing differences in treatment duration and sex ratio, respectively, between the age groups. The Kruskal–Wallis test was used to compare pain intensity and sleeping difficulty by age group. If a significant difference was found, a pair of variables in the three groups was assessed with the Mann–Whitney *U* test. Because three tests were performed, Bonferroni adjustment was applied with the alpha level set at p = 0.0167 (i.e., 0.05/3). The Wilcoxon signed-rank test was used to compare subjective symptoms between the pretreatment and the post-treatment periods. A value of p < 0.05 was considered significant. All analyses were performed by using IBM SPSS Statistics 21 software (IBM Japan, Tokyo, Japan).

## Results

No age group showed any significant sex differences. In general, 84.4% (151/179) of the study population included female patients with a higher proportion in group M3 (88.2%) than in groups M1 (80.5%) or M2 (82.3%). However, the sex ratio was not significantly different between the age groups (chi-square test, p = 0.60) (Table 1).

**Table 1:** **Demographic data of patients with myofascial TMDs by age group**

Variable	M1 (n = 41)	M2 (n = 62)	M3 (n = 76)
Median age (y)	15.5 ± 2.5	29.6 ± 5.9	54.9 ± 10.8
Age range (y)	10 - 19	20 - 39	40 - 84
Female/male ratio	33/8	51/11	67/9

M1 = less than 20 years; M2 = 20–39 years; M3 = 40 years and older.

The data are presented by the mean ± the standard deviation or by the number of patients.

Pretreatment pain intensity was not significantly different among the age groups (Table 2). However, significant differences were observed in sleeping difficulty (Kruskal–Wallis test, p = 0.009). Groups M2 and M3 had similar sleeping difficulties (Mann–Whitney *U* test, p = 0.71), but scored significantly higher than group M1 (Mann–Whitney *U* test: M1 vs. M2, p = 0.006; M1 vs. M3, p = 0.005).

**Table 2:** **Comparison of pretreatment symptom scores between the age groups**

Symptom	M1 (n = 41)	M2 (n = 62)	M3 (n = 76)	p*
Jaw/face pain	5.5 ± 2.9	5.3 ± 2.4	5.5 ± 2.2	0.83
Headache	4.2 ± 3.3	3.6 ± 2.5	4.2 ± 3.3	0.41
Neck pain	3.2 ± 3.4	3.6 ± 2.7	4.1 ± 3.0	0.26
Sleeping difficulty	2.7 ± 3.2	4.2 ± 3.0	4.5 ± 3.4	0.009

M1 = less than 20 years; M2 = 20–39 years; M3 = 40 years and older.

The data are presented by the mean ± the standard deviation.

*The p value is based on the Kruskal–Wallis test.

Regarding treatment options, patients in groups M2 and M3 were more likely to receive pharmacotherapy, compared to patients in group M1 (Table 3). However, no significant difference in the distribution of various treatments was noted (chi-square test, p = 0.76). The average treatment durations were 15.8 weeks, 18.7 weeks, and 20.0 weeks in groups M1, M2, and M3, respectively, but this was not significantly different (ANOVA, p = 0.68).

**Table 3:** **Treatment options and duration by age group**

Treatment	M1 (n = 41)	M2 (n = 62)	M3 (n = 76)
Pharmacotherapy	23 (56.1)	50 (82.0)	52 (83.1)
Physical therapy	25 (61.0)	45 (75.4)	47 (61.0)
Pharmacotherapy + physical therapy	17 (41.5)	38 (62.3)	41 (53.2)
Orthopedic appliances	6 (14.6)	8 (13.1)	7 (9.1)
Outside reference	5 (12.2)	3 (4.9)	9 (11.7)
Duration of treatment (wk)	15.8 ± 10.3	18.7 ± 18.3	20.0 ± 33.2

M1 = less than 20 years; M2 = 20–39 years; M3 = 40 years and older.

The data are presented by the number of patients (%) or by the mean ± the standard deviation.

After treatment, the intensity of jaw/face pain, headache, and sleeping difficulty significantly improved in groups M2 and M3. However, only the intensity of jaw/face pain significantly reduced in group M1 (Table 4). The changes in the scores of pain intensity and sleeping difficulty were not different between the groups (Table 5).

**Table 4:** **Comparison of pretreatment and post-treatment symptom scores by age group**

Symptom	M1 (n = 41)	p*	M2 (n = 62)	p*	M3 (n = 76)	p*
Jaw/face pain						
Before	5.5 ± 2.9	0.002	5.3 ± 2.4	< 0.001	5.5 ± 2.2	< 0.001
After	4.1 ± 2.4		3.5 ± 1.9		3.6 ± 2.4	
Headache						
Before	4.2 ± 3.3	0.28	3.6 ± 2.5	0.002	4.2 ± 3.3	0.001
After	3.6 ± 3.0		2.3 ± 2.1		2.4 ± 2.5	
Neck pain						
Before	3.2 ± 3.4	0.88	3.6 ± 2.7	0.08	4.1 ± 3.0	0.07
After	3.1 ± 2.9		3.0 ± 2.4		3.2 ± 2.5	
Sleeping difficulty						
Before	2.7 ± 3.2	0.96	4.2 ± 3.0	0.001	4.5 ± 3.4	0.005
After	2.7 ± 3.1		2.8 ± 2.6		3.2 ± 3.0	

M1 = less than 20 years; M2 = 20–39 years; M3 = 40 years and older.

The data are presented by the mean ± the standard deviation.

*The p value is based on the Wilcoxon signed-rank test.

**Table 5:** **Comparison of the changes in the scores between age groups**

Symptom	M1 (n = 41)	M2 (n = 62)	M3 (n = 76)	p*
Jaw/face pain	-1.4 ± 2.8	-1.9 ± 2.5	-2.0 ± 3.2	0.53
Headache	-0.5 ± 3.1	-1.3 ± 3.1	-1.8 ± 4.3	0.24
Neck pain	0.1 ± 2.4	-0.5 ± 2.7	-0.8 ± 3.9	0.33
Sleeping difficulty	-0.1 ± 2.8	-1.5 ± 3.2	-1.2 ± 4.7	0.08

M1 = less than 20 years; M2 = 20–39 years; M3 = 40 years and older.

The data are presented by the mean ± the standard deviation. The score change is calculated by the difference between the post-treatment and pretreatment symptom scores (i.e., score change = post-treatment symptom score – pretreatment symptom score).

*The p value is based on the Kruskal–Wallis test.

## Discussion

This study focused on patients with myofascial TMDs and compared self-reported symptoms between three age groups. From the findings of previous studies [5, 26], we hypothesized that young patients are more likely to have mild symptoms and their symptoms are easy to treat, whereas older patients are more likely to show severe symptoms, which are difficult to treat. However, our present findings did not support this hypothesis: pretreatment symptoms of myofascial TMDs were similar in all age groups and no differences were found in the treatment outcomes among the different age groups.

A study of consecutive patients of all ages showed that 85.4% of patients who sought treatment for TMDs were females [27], which was consistent with our findings in each age group (i.e., more than 80%). Therefore, TMDs show a female preponderance at all ages. Temporomandibular disorder conditions such as myofascial pain are associated with female sex [28]. Women with TMDs report more severe physical symptoms, compared to men [29]. However, ratings of pain rarely show significant sex differences [30]. In our study, pain intensity was not significantly different between the sexes, which may have been influenced by the selection bias associated with the patients’ seeking health care [31].

Levitt and McKinney [29] report that the pain severity of patients with TMDs is the same across age groups and that the severity of symptoms is greater in groups in which TMDs have existed for a long duration. We did not study the natural course of the disease in the present study; however, the patients with myofascial TMDs experienced similar pain intensity, regardless of age. From our results, the duration of treatment was not different between the age groups, which suggests a similar duration of TMDs. Furthermore, each age group showed a wide range in the standard deviation for each symptom score, which indicated different levels of severity in each age group. These factors may have influenced the lack of a significant difference in pain intensity associated with myofascial TMDs. However, even patients younger than 20 years have headache and neck pain intensity that is similar to that of older groups. Individuals who develop TMDs are more likely to describe comorbidities such as headache and other body pain [32, 33]. Clinicians should therefore pay more attention to young patients with myofascial TMDs who complain of high-intensity pain.

Myogenous pain is treated by various strategies such as trigger point injections, vapocoolant spray and stretch, transcutaneous electrical nerve stimulation, biofeedback, posture correction, tricyclic antidepressants, muscle relaxants and other medications, and by addressing perpetuating factors [34]. Fricton [34] states that the complexity of the treatment program needs to match the complexity of the patient’s condition. In the current study, pharmacotherapy was provided by one of two board-certified orofacial pain specialists and included analgesics (nonsteroidal anti-inflammatory drugs), muscle relaxants (cyclobenzaprine, 10 mg), and low-dose tricyclic antidepressants (amitriptyline, 10–25 mg). Physical therapy was provided by a licensed therapist and included a home-care program (i.e., self-management and exercise regimen), posture training, mobilization, and the use of physical agents such as ultrasound or vapocoolant spray. Dental treatment other than orthopedic appliances (i.e., interocclusal splints) was not provided to most patients.

The treatment options and duration did not significantly differ by age group, although medications tended to be prescribed more frequently in groups M2 and M3. The average duration of treatment was more varied in group M3. We did not determine the treatment periods of each patient because the treatment interval varied among the age groups. We defined the treatment duration as the time from the first visit to the last visit. Further, older patients may have a chronicity of symptoms [5]. These factors may have influenced the large dispersion of treatment duration in group M3.

A study that compared treatment outcomes of young patients (20–30 years) and elderly patients (50–70 years) with TMDs [18] showed that, although 54% and 38% of the respective groups had a muscle disorder diagnosis, both groups responded equally well to a conservative treatment regimen and experienced marked reduction in pain. In the present study, the patients in all age groups demonstrated a significant decrease in jaw/face pain after the treatments, and the changes in the scores were not different between the age groups. Therefore, conservative treatment methods are effective for myofascial TMDs at all ages.

Approximately one-third of patients with TMDs report poor sleep quality [1]. Numerous factors such as medical condition, mental disorders, breathing disorders during sleep, or other sleep disorders can induce insomnia symptoms [35]. A population-based study reports that insomnia is one consequence of chronic pain [36]. A recent epidemiological study reports that the prevalence of difficulty in maintaining sleep increased with age, reaching nearly 50% in elderly individuals (i.e., older than 60 years) [37]. Older patients are more likely to have a physical illness—especially arthritis and heart disease—or have a painful physical affliction such as back pain. These physical conditions may cause older patients to experience greater sleeping difficulty, compared to young patients. In our study, groups M2 and M3 had greater pretreatment sleeping difficulty but had a significant post-treatment improvement. They also reported a similar level of sleep difficulty as that of the young patients. A meta-analysis of fibromyalgia showed that patients treated with cyclobenzaprine were three times more likely to report moderate reductions in individual symptoms, particularly in sleep [38]. In the present study, medications (i.e., muscle relaxants) and a home-care program (i.e., sleeping position and using appropriate pillows) may have improved self-reported sleep difficulty in the older patients.

We did not assess treatment effectiveness. Because of this limitation, we cannot describe the most effective treatment for symptoms of myofascial TMDs in young or elderly patients. However, tailored treatment protocols are necessary for patients with TMDs. Further well-designed studies are needed to clarify the effects of each treatment and the effects of patient compliance with a home-care program on reducing orofacial pain and difficulty in sleeping.

## Conclusions

Pain intensity associated with myofascial TMDs does not differ by age, but older patients experience greater sleeping difficulty, compared to young patients. Conservative treatment strategies can reduce pain in the jaw or face region at all ages. Treatment outcomes of self-reported pain intensity and sleeping difficulty are not different between different age groups. Clinicians should carefully consider the age-related characteristics of patients with myofascial TMDs when developing appropriate management strategies.

## Abbreviations

TMD: Temporomandibular disorder
TMJ: Temporomandibular joint
RDC/TMD: Research diagnostic criteria for temporomandibular disorders
UCSF: University of California San Francisco
NRS: Numeric rating scale
ANOVA: Analysis of variance.
